# Exploration of Overdose Risk Score and Postoperative Complications and Health Care Use After Total Knee Arthroplasty

**DOI:** 10.1001/jamanetworkopen.2021.13977

**Published:** 2021-06-28

**Authors:** Ahmed K. Emara, Daniel Santana, Daniel Grits, Alison K. Klika, Viktor E. Krebs, Robert M. Molloy, Nicolas S. Piuzzi

**Affiliations:** 1 Department of Orthopaedic Surgery, Cleveland Clinic Foundation, Cleveland, Ohio

## Abstract

**Importance:**

The adverse outcomes after total knee arthroplasty (TKA) associated with preoperative prescription drug use (ie, use of narcotics, sedatives, and stimulants) have been established but are not well quantified.

**Objective:**

To test the association of preoperative overdose risk score (ORS) with postoperative health care use.

**Design, Setting, and Participants:**

This cohort study was conducted using data on a consecutive sample of individuals who underwent primary TKA from November 2018 through March 2020 at a tertiary care health system. Data were collected using the Orthopaedic Minimal Data Set Episode of Care, a validated data-collection system for all elective orthopedic surgical interventions taking place within the health care system. Outcomes were assessed at 90 days postoperatively. Individuals whose preoperative baseline characteristics or ORS were not provided or who declined to participate were excluded. Data were analyzed from September through October 2020.

**Exposure:**

Patient-specific preoperative ORS, as measured using NarxCare, associated with patterns of prescription drug use.

**Main Outcomes and Measures:**

Associations between patient-specific ORS categories and 90-day postoperative health care use (ie, prolonged hospital length of stay [LOS; ie, >2 days], nonhome discharge, all-cause 90-day readmission, emergency department [ED] visits, and reoperation) were evaluated. Outcomes were also compared between a group of individuals with ORS less than 300 vs those with ORS 300 or greater who were propensity score matched (4:1; caliper, 0.1) using demographic characteristics (ie, age, sex, race, body mass index, and smoking status) and baseline comorbidities.

**Results:**

Among 4326 individuals who underwent primary TKA, 2623 (60.63%) were women, 3602 individuals (83.26%) were White, the mean (SD) BMI was 32.8 (6.9), and the mean (SD) age was 66.6 (9.2) years; 90-day follow-up was available for the entire cohort. The predominant preoperative diagnosis was osteoarthritis, occurring among 4170 individuals (96.4%). For individuals with an ORS of 300 to 399, there were significantly higher odds of a prolonged LOS (odds ratio [OR], 2.03; 95% CI, 1.46-2.82; *P* < .001), nonhome discharge (OR, 2.01; 95% CI, 1.37-2.94; *P* < .001), all-cause 90-day readmission (OR, 1.56; 95% CI, 1.01-2.42; *P* < .001), and ED visits (OR, 1.62; 95% CI, 1.11-2.38; *P* = .01) compared with individuals who were prescription drug naive (ie, ORS = 0). Individuals in the highest ORS category (ie, ORS ≥ 500) had the highest ORs for prolonged LOS (OR, 3.71; 95% CI, 2.00-6.87; *P* < .001), nonhome discharge (OR, 4.09; 95% CI, 2.02-8.29; *P* < .001), 90-day readmission (OR, 4.41; 95% CI, 2.23-8.71; *P* < .001), and 90-day reoperation (OR, 6.09; 95% CI, 1.44-25.80; *P* = .01). Propensity score matching confirmed the association between an ORS of 300 or greater and the incidence of prolonged LOS (244 individuals [11.6%] vs 130 individuals [23.0%]; *P* < .001), nonhome discharge (176 individuals [8.4%] vs 93 individuals [16.4%]; *P* < .001), all-cause 90-day readmission (119 individuals [5.7%] vs 65 individuals [11.5%]; *P* < .001), and all-cause ED visits (198 individuals [9.4%] vs 76 individuals [13.4%]; *P* = .006).

**Conclusions and Relevance:**

This study found that higher ORS was associated with increased health care use after primary TKA. These findings suggest that an ORS of 300 or greater could be used to designate increased risk and guide the preoperative surgeon-patient discussion to modify prescription drug use patterns.

## Key Points

**Question:**

Is the preoperative overdose risk score associated with postoperative complications and health care use after total knee arthroplasty?

**Findings:**

In this cohort study of 4326 individuals who underwent primary total knee arthroplasty, those with a preoperative overdose risk score of 300 or greater had statistically significantly higher odds of prolonged hospital length of stay, nonhome discharge, all-cause 90-day readmission, and emergency department visits.

**Meaning:**

These findings suggest that overdose risk score could be used to identify high-risk prescription drug use patterns before primary total knee arthroplasty and to counsel such individuals to modify their drug use patterns to avert adverse postoperative outcomes.

## Teaser

This cohort study investigates the association of an overdose risk score with postoperative complications and health care use after total knee arthroplasty.

## Introduction

The US health care system is under strain from substance use.^1,2,3^ In 2018, approximately 6.2% of the population self-reported abusing at least 1 type of prescription drug, including stimulants, sedatives, and opioids.^4^ While total knee arthroplasty (TKA) has been found to be associated with safe and effective improvements in functional outcomes even among individuals with underlying comorbidities,^5^ contemporary literature indicates a significant association between preoperative substance use and post-TKA adverse outcomes.^6,7,8,9,10^ Preoperative use of opioids, stimulants, sedatives, or inhalants has been associated with significantly longer hospital length of stay (LOS), 8-fold the odds of leaving against medical advice, and a 5-fold increase in the incidence of postoperative mortality.^10^ Despite the grave implications, a quantitative assessment and a consequent high-risk designation threshold based on combined controlled substance use (including narcotics, sedatives, and stimulants) have not been established.^8,11,12^

While preoperative prescription drug use status has been assessed as a qualitative risk factor,^7,13,14^ finding a quantitative association between prescription drug use and adverse outcomes after TKA has been impeded by the lack of reliable and readily available data regarding patient-specific prescription drug use. This limitation has hindered in-depth analyses within contemporary literature, including studies evaluating the association between the most commonly used prescription drugs (ie, opioids) and post-TKA outcomes. In a recent meta-analysis, Goplen et al^15^ reported that parameters for designating preoperative opioid use ranged from any documented opioid use within 2 years before the index surgical treatment to a minimum of 6 weeks of opioid use prior to the index procedure. However, investigations that described dose-based designations used a minimum preoperative dose of more than 20 mg or 30 mg milliequivalents. Therefore, the lack of a quantitative assessment that incorporates dose, duration, and pattern of prescription drug consumption has posed a consistent limitation to translating literature findings to clinical practice.^16^

The nescience of a quantitative association between pre-TKA substance use and postoperative outcomes warrants an exploration of computable, readily available modalities that can be consistently applied in clinical settings. The overdose risk score (ORS) was calculated using a quantifiable reflection of prescription drug monitoring program data regarding patient-specific prescription drug use (sedatives, stimulants, and opioids combined) that is currently integrated into individuals’ electronic medical records across 43 states in the US and supported by the Health Information Technology for Economic and Clinical Health Act.^17,18,19,20,21,22^ This study aimed to characterize the association between preoperative ORS as a measure of individuals’ prescription drug use and 90-day postoperative readmissions, emergency department (ED) visits, reoperation, prolonged (ie, >2 days) LOS, and nonhome discharge. In addition, we sought to evaluate this quantitative association by outlining an ORS threshold beyond which individuals would be considered at moderate or high risk of adverse outcomes.

## Methods

The Cleveland Clinic Foundation's institutional review board approval was obtained for this cohort study. The study followed the Strengthening the Reporting of Observational Studies in Epidemiology (STROBE) reporting guideline, and all patients provided informed consent prior to enrollment.

### Study Design and Setting

A prospectively collected cohort of all individuals who received primary TKA from 1 of 27 surgeons at an integrated North American health care system from November 2018 through March 2020 was retrospectively reviewed. The study cohort was obtained using a validated institutional prospective data-collection system (Orthopaedic Minimal Data Set Episode of Care [OME]) that has been previously described and validated.^23,24,25^ The OME records data-collection system captures more than 97% of orthopedic elective surgical interventions within the health care system and records patient demographic characteristics, baseline comorbidities, in-hospital metrics (ie, surgical details, LOS, and discharge disposition), readmission, and ED visits up to 90 days postoperatively, as well as reoperation and mortality up to 1 year postoperatively.

### Admission ORS

Updated ORSs were extracted from the included individuals’ electronic medical records at the time of index surgical admission. These scores use the NarxCare platform (Appriss Health) to query the prescription drug monitoring program at each patient encounter and are numerical scores ranging from 0 (ie, individuals who are prescription drug naive) to 999, with higher scores indicating a greater risk of prescription drug overdose.^18,19,20^ This numerical score is obtained through an algorithm that analyzes current and past prescription drug use (ie, use of opioids, sedatives, and stimulants) from the prescription drug monitoring program and accounts for dose (in milligram equivalences), prescription overlap, and number of prescribing clinicians and dispensing pharmacies.^18,20^ Therefore, the ORS is a reliable, routinely available, quantitative reflection of individuals’ overall consumption of prescription drugs that also accounts for prescription and dispensation patterns to assess patient-specific risk of use and overdose.

### Study Population

Among 4567 individuals who received unilateral primary elective TKA within the study period, all were considered eligible for inclusion. Of these, 241 individuals (5.3%) were excluded, including 188 individuals (4.1%) excluded owing to unavailable ORSs preoperatively and 53 individuals (1.2%) owing to incomplete or undisclosed race or sex.

### Outcomes of Interest

This study’s primary outcome was 90-day postoperative health care use, including prolonged LOS (ie, >2 days), nonhome discharge, all-cause 90-day readmission, ED visits, and reoperation. Secondary outcomes included the occurrence of procedure-related and non–procedure-related 90-day readmission, as stratified by Schairer et al.^26^ The occurrence of medical, surgical, and pain-related ED visits was also analyzed. Associations between patient-specific ORS category and these outcomes were evaluated. In addition, the association between ORS as a continuous variable and the outcomes of interest was assessed using spline regression models.

### Statistical Analysis

**Figure 1.**
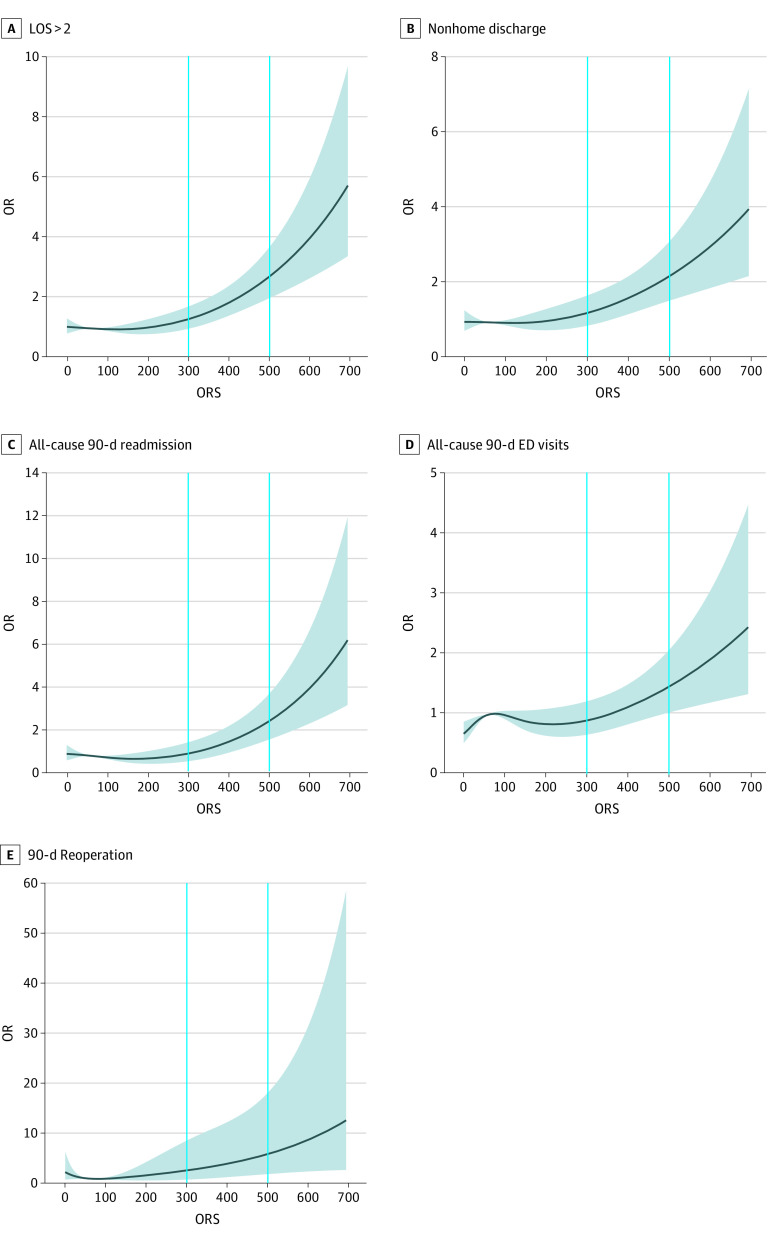
Spline Regression Model for Association Between Overdose Risk Score (ORS) and Primary Outcomes ED indicates emergency department; LOS, hospital length of stay; OR, odds ratio.

**Figure 2.**
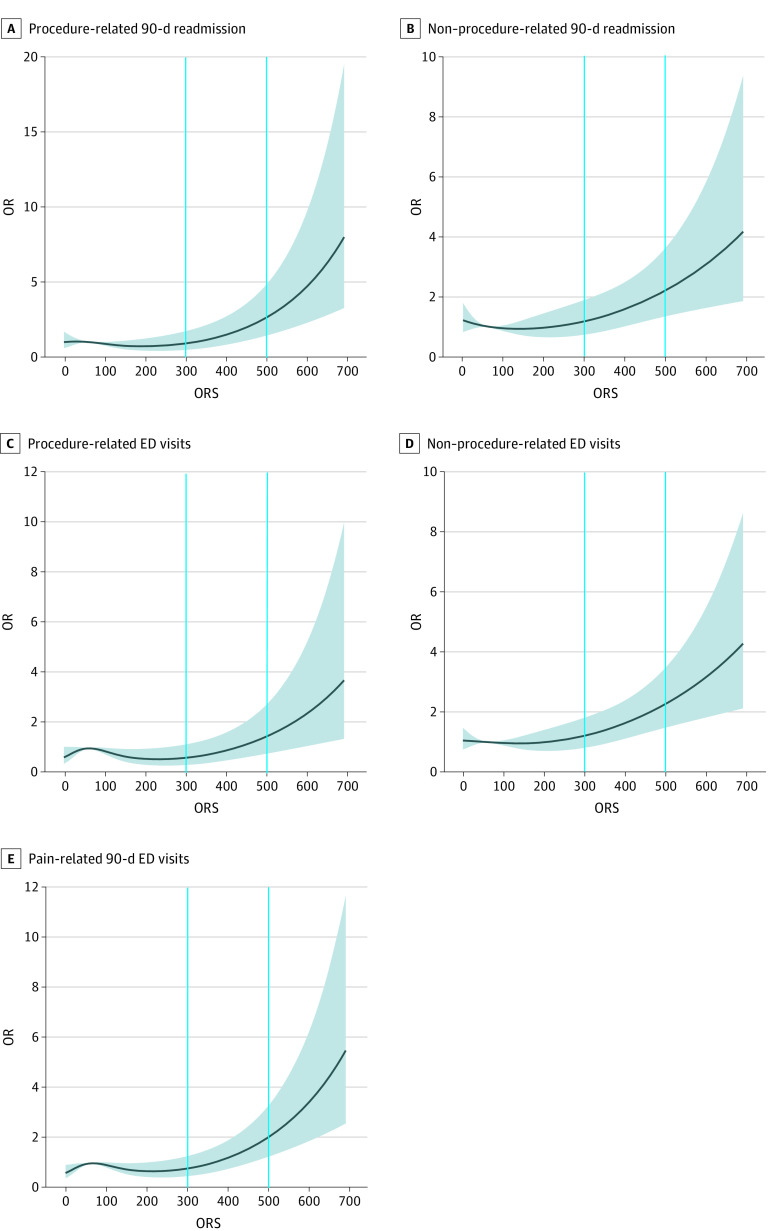
Spline Regression Model for Association Between Overdose Risk Score (ORS) and Secondary Outcomes ED indicates emergency department; OR, odds ratio.

Bivariate analysis was performed to outline the distribution of individual demographic characteristics, comorbidities, and outcomes within the included cohort. Descriptive statistics were computed for ORS as a continuous variable (as mean [SD]) for the evaluated risk factors and outcomes. Individuals were stratified by preoperative ORS into 6 groups: those with an ORS of 0 (ie, individuals who were prescription drug naive), 1 through 99, 100 through 199, 200 through 299, 300 through 399, 400 through 499, and 500 or greater.^18,19,20^ Multivariable logistic regression was performed to assess independent associations between ORS category and outcomes while adjusting for confounders, including age group, sex, race, body mass index (BMI; calculated as weight in kilograms divided by height in meters squared) category, smoking status, and baseline comorbidities (using the Charlson Comorbidity Index [CCI]^27^) (eTables 1-9 in the Supplement). Age, race, and sex were self-reported, and BMI was investigator assessed. These variables were captured and accounted for because they have been previously established as independent risk factors and potential confounders associated with the outcomes of interest. The lowest ORS category that was associated with statistically significantly higher odds of developing the primary outcomes (ie, 300) was designated as a high-risk threshold. To confirm the association, a nearest-neighbor 4:1 propensity score–matched comparison was conducted between all outcomes among 2106 individuals with ORSs below the threshold and 566 individuals with ORSs equal to or greater than the threshold. The purpose of this propensity score–matched comparison was to verify the association through an additional quasirandomization process that accounted for potential confounders (ie, age, sex, race, BMI, smoking status, and CCI score category) (eTable 10 in the Supplement). A matching caliper of 0.1 was used. Plots of the spline regression models were created using a restricted cubic spline curve with 4 knots (Figure 1 and Figure 2). These plots graphed the associations between ORS as a continuous variable and the outcomes of interest. Odds ratios (ORs; odds of a specific outcome per operative time compared with median operative time) were used in the plots to aid interpretation. All tests were 2-sided, and the α level was set at .05, with the significance level at *P* < .05. Statistical analyses were completed using R statistical software version 3.4.1 (R Project for Statistical Computing).

## Results

Among 4326 individuals with complete baseline data who were subsequently analyzed, 2623 (60.63%) were women, 3602 individuals (83.26%) were White, the mean (SD) age was 66.6 (9.2) years, and the mean (SD) BMI was 32.8 (6.9). The mean (SD) ORS was 117.9 (139.3), with 1440 individuals (33.3%) in the ORS 0 category and 1066 individuals (24.6%) in the ORS 1 through 99 category (Table 1; eTable 11 in the Supplement). Among all individuals, 283 (6.54%) were readmitted. The most common preoperative diagnosis was osteoarthritis, which was found among 4170 individuals (96.4%) (eTable 12 in the Supplement). Of the included population, there was a history of use opioids among 2856 individuals (66.0%), sedatives among 2856 individuals (66.0%), and stimulants among 149 individuals (3.4%) (eTables 13-15 in the Supplement).

**Table 1.** Sample Characteristics and ORS by Individuals’ Demographics, Baseline Comorbidities, and Outcomes

Variable	Individuals, No. (%) (N = 4326)	ORS, mean (SD)	*P* value^a^
**Risk factor**			
Age group, y			
18-29	4 (0.09)	165.00 (98.15)	<.001
30-39	23 (0.53)	198.17 (170.90)	
40-49	124 (2.87)	147.16 (151.92)	
50-59	775 (17.91)	133.74 (153.47)	
60-69	1702 (39.34)	126.51 (144.50)	
70-79	1385 (32.02)	99.86 (122.84)	
80-89	311 (7.19)	94.12 (121.51)	
≥90	2 (0.05)	90.00 (0)	
Sex			
Women	2623 (60.63)	124.52 (142.28)	<.001
Men	1703 (39.37)	107.80 (134)	
Race			
White	3602 (83.26)	116.77 (138.93)	.20
Black	544 (12.58)	121.33 (139.40)	
Asian	38 (0.88)	86.84 (92.77)	
American Indian or Alaska Native	9 (0.21)	161.11 (109.25)	
Multiracial or multicultural	66 (1.53)	146.20 (168.78)	
BMI classification			
Underweight, BMI <18.5	3 (0.07)	46.67 (80.83)	.09
Reference range weight, BMI 18.5-24.9	392 (9.06)	111.38 (143.16)	
Overweight, BMI 25.0-29.9	1234 (28.53)	110.53 (135.50)	
Obese			
Class I, BMI 30.3-34.9	1237 (28.59)	118.54 (138.21)	
Class II, BMI 35.0-39.9	857 (19.81)	124.74 (144.24)	
Class III, BMI ≥40.0	603 (13.94)	126.78 (139.17)	
Smoking status			
Never	2393 (55.32)	109.15 (133.38)	<.001
Quit			
>6 mo	1509 (34.88)	118.73 (137.23)	
<6 mo	116 (2.68)	135.46 (160.86)	
Current	308 (7.12)	175.68 (168.83)	
CCI score category			
0-2 (low risk)	3054 (70.6)	112.98 (135.68)	<.001
3-4 (moderate risk)	1137 (26.28)	130.70 (148.35)	
≥5 (high risk)	135 (3.12)	122.57 (135.75)	
**90-d outcome**			
LOS, d			
≤2	3762 (86.96)	112.87 (133.64)	<.001
>2	564 (13.04)	151.70 (168.68)	
Discharge disposition			
Home	3908 (90.34)	114.79 (136.10)	<.001
Nonhome	418 (9.66)	147.35 (163.57)	
All-cause 90-d readmission			
No	4043 (93.46)	115.90 (136.19)	<.001
Yes	283 (6.54)	147.05 (175.69)	
Procedure-related readmission			
No	4229 (97.76)	117.06 (137.95)	.01
Yes	97 (2.24)	155.88 (186.14)	
Non–procedure-related readmission			
No	4135 (95.58)	117.08 (137.78)	.06
Yes	191 (4.42)	136.35 (168.47)	
90-d ED visit			
No	3881 (89.71)	115.86 (137.87)	.004
Yes	445 (10.29)	136.05 (150.25)	
Procedure-related ED visit			
No	4223 (97.62)	117.45 (138.65)	.15
Yes	103 (2.38)	137.66 (163.43)	
Non–procedure-related ED visit			
No	4070 (94.08)	116.20 (136.98)	<.001
Yes	256 (5.92)	145.46 (170.14)	
Pain-related ED visit			
No	4166 (96.3)	116.33 (137.18)	<.001
Yes	160 (3.7)	159.78 (181.88)	
90-d Reoperation			
No	4293 (99.24)	117.53 (138.82)	.03
Yes	33 (0.76)	170.79 (188.06)	

Abbreviations: BMI, body mass index (calculated as weight in kilograms divided by height in meters squared); CCI, Charlson Comorbidity Index; ED, emergency department; LOS, hospital length of stay; ORS, overdose risk score.

^a^ *P* values were calculated by 2-sample *t* test or analysis of variance depending on the number of categories.

### Primary Outcomes

In multivariable regression analysis, there were statistically significantly higher odds of prolonged LOS (OR, 2.03; 95% CI, 1.46-2.82; *P* < .001), nonhome discharge (OR, 2.01; 95% CI, 1.37-2.94; *P* < .001), all-cause 90-day readmission (OR, 1.56; 95% CI, 1.01-2.42; *P* = .045), and ED visits (OR, 1.62; 95% CI, 1.11-2.38; *P* = .01) among individuals in the ORS 300 to 399 category compared with individuals who were prescription drug naive (Table 2). The ORs for such adverse outcomes were higher for individuals in the ORS 400 to 499 category compared with those in the 300 to 399 category (prolonged LOS: OR, 3.04; 95% CI, 2.06-4.49; *P* < .001; nonhome discharge: OR, 3.16; 95% CI, 2.02-4.92; *P* < .001; all-cause 90-day readmission: OR, 2.04; 95% CI, 1.22-3.39; *P* = .006; ED visits: OR, 2.04; 95% CI, 1.29-3.21; *P* = .002). Individuals in the ORS 500 or greater group had the highest ORs for prolonged LOS (OR, 3.71; 95% CI, 2.00-6.87; *P* < .001), nonhome discharge (OR, 4.09; 95% CI, 2.02-8.29; *P* < .001), and 90-day readmission (OR, 4.41; 95% CI, 2.23-8.71; *P* < .001). Notably, individuals in this category did not have statistically significantly higher odds of 90-day ED visits compared with individuals who were prescription drug naive. Conversely, the ORS 500 or greater category was the only group with statistically significantly higher odds of 90-day reoperation (OR, 6.09; 95% CI, 1.44-25.80; *P* = .01). Restricted cubic spline models further outlined the association between ORS as a continuous variable and the corresponding odds of experiencing each of the primary outcomes, as demonstrated in Figure 1. Therefore, in our stratified assessment of individual-specific ORs, we found that among individuals who were not prescription drug naive (ie, previous use reported, as indicated by ORS > 0), only individuals with an ORS of 300 or greater had clinically and statistically significantly higher odds of adverse outcomes; this corresponds to 575 of 2886 individuals (19.9%) with any documented prescription drug use.

**Table 2.** Odds of Primary and Secondary Outcomes by ORS Category After Adjustment^a^

ORS category	LOS > 2		Nonhome discharge		Readmission		ED visit		Reoperation	
	OR (95% CI)	*P* value	OR (95% CI)	*P* value	OR (95% CI)	*P* value	OR (95% CI)	*P* value	OR (95% CI)	*P* value
0	1 [Reference]	NA	1 [Reference]	NA	1 [Reference]	NA	1 [Reference]	NA	1 [Reference]	NA
1-99	0.996 (0.771-1.288)	.98	0.975 (0.724-1.312)	.87	0.959 (0.688-1.337)	.81	1.478 (1.124-1.942)	.005	0.516 (0.177-1.503)	.23
100-199	0.881 (0.658-1.179)	.39	1.061 (0.768-1.466)	.72	0.776 (0.524-1.147)	.2	1.424 (1.056-1.92)	.02	0.472 (0.13-1.718)	.26
200-299	0.937 (0.662-1.326)	.71	0.765 (0.498-1.174)	.22	0.623 (0.373-1.04)	.07	0.949 (0.643-1.399)	.79	0.742 (0.203-2.714)	.65
300-399	2.027 (1.456-2.821)	<.001	2.006 (1.371-2.937)	<.001	1.563 (1.01-2.421)	.045	1.62 (1.105-2.375)	.01	1.386 (0.426-4.51)	.59
400-499	3.038 (2.058-4.485)	<.001	3.155 (2.023-4.919)	<.001	2.036 (1.224-3.387)	.006	2.035 (1.288-3.214)	.002	1.342 (0.355-5.082)	.67
≥500	3.71 (2.002-6.873)	<.001	4.091 (2.018-8.294)	<.001	4.408 (2.232-8.707)	<.001	1.77 (0.843-3.714)	.13	6.093 (1.439-25.801)	.01
	**Non–procedure-related cause of readmission**		**Procedure-related cause of readmission**		**Non–procedure-related cause of ED visit**		**Procedure-related cause of ED visit**		**Pain-related ED visit**	
0	1 [Reference]	NA	1 [Reference]	NA	1 [Reference]	NA	1 [Reference]	NA	1 [Reference]	NA
1-99	0.862 (0.578-1.285)	.47	1.126 (0.656-1.932)	.67	1.043 (0.734-1.482)	.82	1.378 (0.816-2.328)	.23	1.2 (0.765-1.882)	.43
100-199	0.81 (0.515-1.274)	.36	0.61 (0.294-1.264)	.18	0.943 (0.633-1.405)	.77	1.01 (0.544-1.874)	.98	1.34 (0.831-2.16)	.20
200-299	0.548 (0.292-1.031)	.06	0.708 (0.307-1.635)	.42	0.861 (0.526-1.409)	.55	0.592 (0.243-1.442)	.25	0.591 (0.283-1.23)	.20
300-399	1.465 (0.873-2.459)	.15	1.458 (0.692-3.071)	.32	1.727 (1.095-2.723)	.02	1.098 (0.496-2.43)	.82	1.487 (0.812-2.725)	.20
400-499	1.777 (0.959-3.291)	.07	1.838 (0.78-4.335)	.16	2.072 (1.203-3.567)	.009	1.915 (0.811-4.525)	.14	2.23 (1.152-4.316)	.02
≥500	2.663 (1.071-6.623)	.04	5.977 (2.366-15.098)	<.001	2.793 (1.246-6.262)	.01	3.545 (1.196-10.51)	.02	4.89 (2.123-11.262)	<.001

Abbreviations: ED, emergency department; LOS, hospital length of stay; NA, not applicable; OR, odds ratio; ORS, overdose risk score.

^a^ Adjusted for age, sex, race, body mass index, smoking status, and baseline comorbidities through the Charlson Comorbidity Index.

### Secondary Outcomes

After stratification of 90-day readmission by cause, we found that individuals in the ORS 500 or greater category had statistically significantly higher odds of procedure-related 90-day admission (OR, 5.98; 95% CI, 2.37-15.1; *P* < .001) and non–procedure-related 90-day admission (OR, 2.66; 95% CI, 1.07-6.62; *P* = .04) (Table 2; Figure 2). The lowest ORS category with statistically significantly higher odds of non–procedure-related ED visits was the ORS 300 to 399 category (OR, 1.73; 95% CI, 1.1-2.72; *P* = .02), and the highest OR was in the ORS 500 or greater category (OR, 2.79; 95% CI, 1.25-6.26; *P* = .01). Pain-related ED visits were more likely to occur in the ORS 400 to 499 category (OR, 2.23; 95% CI, 1.15-4.32; *P* = .02) and ORS 500 or greater category (OR, 4.89; 95% CI, 2.12-11.26; *P* < .001), while procedure-related ED visits had statistically significantly higher odds of occurring among individuals in the ORS 500 or greater category (OR, 3.55; 95% CI, 1.2-10.51; *P* = .02).

### Propensity Score–Matched Comparison of Individuals Below vs at or Above ORS Threshold

Compared with individuals with an ORS of less than 300, those at or beyond the 300 threshold had statistically significantly increased incidence of prolonged LOS (244 individuals [11.6%] vs 130 individuals [23.0%]; *P* < .001), nonhome discharge (176 individuals [8.4%] vs 93 individuals [16.4%]; *P* < .001), 90-day all-cause readmission (119 individuals [5.7%] vs 65 individuals [11.5%]; *P* < .001), procedure-related readmission (49 individuals [3.2%] vs 23 individuals [4.1%]; *P* = .019), non–procedure-related readmission (77 individuals [3.7%] vs 31 individuals [7.2%]; *P* < .001), all-cause ED visits (198 individuals [9.4%] vs 76 individuals [13.4%]; *P* = .006), pain-related ED visits (72 individuals [3.4%] vs 36 individuals [6.4%]; *P* = .002), and non–procedure-related ED visits (101 individuals [4.8%] vs 55 individuals [9.7%]; *P* < .001) (Table 3). However, an ORS of 300 or greater was not associated with procedure-related ED visits or 90-day reoperation. Restricted cubic spline models further outlined the association between ORS as a continuous variable and the corresponding odds of experiencing secondary outcomes (Figure 2).

**Table 3.** Propensity Score–Matched Comparison of Outcomes by Overdose Risk Threshold

Outcome	Individuals by overdose risk score, No. (%)		*P* value
	<300 (n = 2106)	≥300 (n = 566)	
LOS > 2	244 (11.6)	130 (23.0)	<.001
Nonhome discharge	176 (8.4)	93 (16.4)	<.001
90-d readmission			
All cause	119 (5.7)	65 (11.5)	<.001
Nonprocedure related	77 (3.7)	31 (7.2)	<.001
Procedure related	49 (3.2)	23 (4.1)	.02
90-d ED visit			
All cause	198 (9.4)	76 (13.4)	.01
Nonprocedure related	101 (4.8)	55 (9.7)	<.001
Procedure related	53 (2.5)	18 (3.2)	.47
Pain related	72 (3.4)	36 (6.4)	.002
90-d reoperation	18 (0.9)	9 (1.6)	.19

Abbreviations: ED, emergency department; LOS, hospital length of stay.

## Discussion

The implications of the opioid epidemic for health care provision, specifically TKA, have been established in contemporary literature.^6,7,14^ Conversely, the outcomes associated with nonopioid prescription medications, including sedatives and stimulants, have not been adequality characterized despite a marked prevalence of misuse.^4^ The lack of a quantitative description of such association within published investigations hinders data-driven patient-specific risk assessment in routine clinical settings.^16^ This cohort study provided a comprehensive quantitative analysis of the association between preoperative prescription drug use patterns and post-TKA adverse outcomes. The ORs of adverse outcomes invariably increased with higher ORS categories. Individuals with an ORS of 300 to 399 were more likely to experience prolonged LOS, nonhome discharge, all-cause 90-day readmission, and ED visits. As ORS increased, individuals had higher ORs of 90-day reoperation, procedure-related and non–procedure-related readmission and ED visits, and pain-related ED visits, with maximal ORs found in the ORS 500 or greater category.

The ORs of adverse outcomes increased with higher ORS categories, which are reflective of the individual’s prescription drug doses and pattern and duration of use. Jain et al^8^ attempted to outline the association between time-quantified pre-TKA opioid consumption and postoperative adverse outcomes through stratifying a cohort of 137 076 individuals according to preoperative duration of opioid use (ie, ≤3 months, >3-6 months, >6 months continuously, and >6 months with cessation at 3 months preoperatively). The authors found that individuals who consumed opioids for 3 months or less preoperatively had equivalent 90-day risk of all-cause ED visits, pain-related ED visits, wound complications, readmission, and reoperation compared with individuals who were opioid naive. A similar pattern was found among the 3-month to 6-month opioid use category, with the exception of a higher 90-day readmission rate compared with individuals who were opioid naive (hazard ratio, 1.54; 95% CI, 1.02-2.33). Opioid use for more than 6 months was associated with an increased risk in all aforementioned adverse outcomes compared with an opioid-naive status; however, opioid cessation 3 months preoperatively was associated with a decrease in the risk of adverse outcomes and a local wound complications risk that was near the reference range compared with individuals who were opioid naive. Similarly, Wilson et al^28^ investigated health care use after revision TKA among individuals who were opioid naive vs those with variable dose and duration of preoperative opioid use. The authors found that continuous preoperative use was associated with higher odds of extended LOS, nonhome discharge, 90-day readmission, and ED visits. In addition, providing a 6-month preoperative opioid holiday was associated with lower odds of these outcomes. These findings align with those of our study in emphasizing the critical role of quantifying substance use rather than using a dichotomous categorization (ie, individuals who are using vs not using or chronically using vs not chronically using).

The inclusion of ORSs in electronic health records is a recent trend, with widespread implementation occurring only in 2017.^17,18,29^ Therefore, literature evaluating the role of ORS as a factor associated with orthopedic-related outcomes is scarce. In a 2019 investigation, Galivanche et al^20^ retrospectively analyzed the association between individuals’ ORS categories (ie, 0, 1-99, 100-299, 300-499, and ≥500) and risk of 30-day readmission, reoperation, and mortality among 346 individuals who received an elective spine surgical procedure. In contrast to our study, the authors reported no association between membership in any ORS category and measured outcomes. However, the authors disclosed that their analysis was limited by sample size, which may have precluded the detection of associations. Furthermore, differences in the nature of the surgical intervention (ie, elective spine surgical procedure vs total knee arthroplasty) may have contributed, in part, to the dissimilarity between the authors’ findings and those of our study. Of note, in the propensity score–matched comparison, our study found higher odds of adverse outcomes in the ORS 300 or greater group for most evaluated outcomes. There was a lack of significant difference in our study in the incidence of procedure-related 90-day ED visits and 90-day reoperation between the ORS less than 300 and ORS 300 or greater groups despite the higher odds of these outcomes within the ORS 500 or greater group vs the ORS 0 group, as found in the logistic regression models. Such a pattern suggests the importance of analyzing prescription drug use in a graduated, quantitative fashion and avoiding analyses that are limited to dichotomous associations.

Our study included all individuals with available ORSs who received primary unilateral TKA performed by 1 of 27 surgeons in a North American health care system. In addition, eligibility criteria did not preclude individuals’ inclusion based on preoperative diagnosis, and the constructed models accounted for potential confounders, including age, sex, race, smoking status, and underlying comorbidities. Therefore, the results of this investigation may be generalizable to most individuals who receive primary unilateral TKA. Nevertheless, further subgroup-dedicated analyses may be warranted to explore the association between ORS and certain preoperative diagnoses of lower-prevalence hardware failure, femoroacetabular impingement, and underlying oncologic pathologies.

Of note, our results associating certain prescription drug use patterns with higher odds of postoperative adverse outcomes may serve to identify individuals at increased risk of adverse postoperative outcomes and trigger a discussion of potential risks. Risk stratification should in no way be used to deem certain individuals ineligible for surgical intervention or to defer surgical treatment based solely on preoperative ORS. In our stratified assessment of individual-specific ORs, we found that among individuals who were not prescription drug naive (ie, previous use reported), only those with an ORS of 300 or greater had clinically and statistically significantly higher odds of adverse outcomes. This corresponds to 575 of 2886 individuals (19.9%) with documented prescription drug use who might otherwise be placed in a single group of individuals who use prescription drugs by a nondiscriminating observer. Therefore, the presented ORS-based odds stratification may serve to guide the preoperative discussion and prompt enhanced counseling and interdisciplinary interventions among individuals with higher ORS rather than denying such individuals surgical intervention.

### Limitations

The findings of this study should be viewed in the context of its limitations. Of 4567 TKAs performed within the study period, 5.3% were excluded owing to the absence of preoperative ORS or critical demographic determinants (ie, undisclosed race, sex, or smoking status). However, complete data were available for 4326 individuals (94.7%), which may limit the risk of selection bias. The group with ORS 500 or greater had the lowest number of individuals, which may have imposed sample size–related limitations.^30^ This may explain the lack of association found between membership in the ORS 500 or greater category and the occurrence of all-cause 90-day ED visits. However, a clear trend of increasing ORs of adverse outcomes was notable with increasing ORS; the spline regression models further supported such a trend. Because 90-day readmission and ED visits were captured in the health care system, some readmissions and ED visits outside of the health care system may have occurred yet remained uncaptured. However, the overall readmission rates reported within this study (6.54%) were similar to those described in the general US Medicare population (6.4%).^31^ Our health care system spans more than 6 centers covering the northeast Ohio region, rendering systematic failure to document readmission unlikely. This was a nonrandomized retrospective investigation, which makes it prone to confounding. However, all analyses were multivariable and accounted for baseline demographic characteristics, as well as underlying comorbidities. Furthermore, propensity score–matched comparison was conducted for a quasirandomized assessment. However, it is noteworthy that this study did not incorporate individuals’ socioeconomic status, insurance status, or psychiatric comorbidities into the multivariable models, which may introduce a degree of confounding bias. We believe the impact of such bias is unlikely to alter the significance of our findings. This study used restricted cubic spline and multivariable regression models but not survival analyses. Such analysis was restricted by the immediate postoperative nature of the evaluated outcomes, particularly LOS and discharge disposition. Future studies with additional follow-up are warranted to explore the association between ORS and the incidence of adverse outcomes at longer follow-ups, as well as any potential association between score values and the time to complication occurrence. While drug dose is weighed heavily in ORS, it is not the sole driver of the score’s final value. In addition, we assessed the overall ORS regardless of the drug-specific ORS subscore values. While such analysis provides value by affording a scalar, readily available, patient-specific assessment of prescription drug use, different drug subtypes may have different complication profiles, and individuals with high-risk ORS may have different variations of drug-specific ORS subscore combinations. For example, an ORS of 500 for an individual with sedative-predominant prescription could be associated with different outcomes compared with a similar individual with an ORS of 500 but an opioid-predominant prescription drug history. Therefore, further investigations are warranted into the association between each of the ORS-weighted elements (ie, dose, pharmacies, prescribing clinicians, and number and overlap of prescriptions), as well as drug-specific ORS subscore and trends of postoperative change in total and drug-specific ORS and postoperative adverse outcomes. Such analyses may reveal potential differences in the weights by which each of these factors contributes to the association described in the present study.

## Conclusions

As elective surgical treatment continues advancing toward patient-specific risk modeling and patient-centered care, a detailed scalar characterization of preoperative risk factors, particularly prescription drug use, is crucial. This study found an association between preoperative ORS and adverse postoperative outcomes. The lowest ORS with such an association was 300, and the ORs of this association peaked among individuals with ORS 500 or greater, among whom there was up to a 6-fold increase in the odds of certain adverse outcomes compared with individuals who were prescription drug naive. These findings suggest that an ORS of 300 may be used as a threshold that would warrant preoperative counseling regarding prescription drug use patterns and associated risks. While higher ORSs may be associated with higher odds of adverse postoperative outcomes, our findings suggest that such scores should prompt a surgeon-patient discussion and an interdisciplinary approach to mitigate deleterious prescription drug use patterns rather than being used as indicators for surgical ineligibility.

## Notes

Supplement. **eTable 1.** Multivariate Regression Demonstrating Odds of Length of Stay >2 Days by Overdose Risk Score Category **eTable 2.** Multivariate Regression Demonstrating Odds of Nonhome Discharge Disposition by Overdose Risk Score Category **eTable 3.** Multivariate Regression Demonstrating Odds of All-Cause 90-Day Readmission by Overdose Risk Score Category **eTable 4.** Multivariate Regression Demonstrating Odds of All-Cause 90-Day Emergency Department Visits by Overdose Risk Score Category **eTable 5.** Multivariate Regression Demonstrating Odds of Procedure-Related 90-Day Readmission by Overdose Risk Score Category **eTable 6.** Multivariate Regression Demonstrating Odds of Non–Procedure-Related 90-Day Readmisison by Overdose Risk Score Category **eTable 7.** Multivariate Regression Demonstrating Odds of Procedure-Related 90-Day Emergency Department Visits by Overdose Risk Score Category **eTable 8.** Multivariate Regression Demonstrating Odds of Non–Procedure-Related 90-Day Emergency Department Visits by Overdose Risk Score Category **eTable 9.** Multivariate Regression Demonstrating Odds of Pain-Related 90-Day Emergency Department Visits by Overdose Risk Score Category **eTable 10.** Distribution of Baseline Determinants Among Overdose Risk Score <300 vs ≥300 Groups After Propensity Score Matching **eTable 11.** Distribution of Included Individuals by Overdose Risk Score Category **eTable 12.** Distribution of Preoperative Diagnoses Within Included Cohort **eTable 13.** Distribution Individual Determinants and Outcomes by Opioid-Specific Overdose Risk Score Category **eTable 14.** Distribution of Individual Determinants and Outcomes by Sedative-Specific Overdose Risk Score Category **eTable 15.** Distribution of Individual Determinants and Outcomes by Stimulant-Specific Overdose Risk Score Category
