# Supplementary material for: Exploration of Overdose Risk Score and Postoperative Complications and Health Care Use After Total Knee Arthroplasty
Source: JAMA Netw Open. 2021 Jun 28;4(6):e2113977. doi: 10.1001/jamanetworkopen.2021.13977 (PMC8239962; doi:10.1001/jamanetworkopen.2021.13977)
Supplement: Supplement. — eTable 1. Multivariate Regression Demonstrating Odds of Length of Stay >2 Days by Overdose Risk Score Category eTable 2. Multivariate Regression Demonstrating Odds of Nonhome Discharge Disposition by Overdose Risk Score Category eTable 3. Multivariate Regression Demonstrating Odds of All-Cause 90-Day Readmission by Overdose Risk Score Category eTable 4. Multivariate Regression Demonstrating Odds of All-Cause 90-Day Emergency Department Visits by Overdose Risk Score Category eTable 5. Multivariate Regression Demonstrating Odds of Procedure-Related 90-Day Readmission by Overdose Risk Score Category eTable 6. Multivariate Regression Demonstrating Odds of Non–Procedure-Related 90-Day Readmisison by Overdose Risk Score Category eTable 7. Multivariate Regression Demonstrating Odds of Procedure-Related 90-Day Emergency Department Visits by Overdose Risk Score Category eTable 8. Multivariate Regression Demonstrating Odds of Non–Procedure-Related 90-Day Emergency Department Visits by Overdose Risk Score Category eTable 9. Multivariate Regression Demonstrating Odds of Pain-Related 90-Day Emergency Department Visits by Overdose Risk Score Category eTable 10. Distribution of Baseline Determinants Among Overdose Risk Score <300 vs ≥300 Groups After Propensity Score Matching eTable 11. Distribution of Included Individuals by Overdose Risk Score Category eTable 12. Distribution of Preoperative Diagnoses Within Included Cohort eTable 13. Distribution Individual Determinants and Outcomes by Opioid-Specific Overdose Risk Score Category eTable 14. Distribution of Individual Determinants and Outcomes by Sedative-Specific Overdose Risk Score Category eTable 15. Distribution of Individual Determinants and Outcomes by Stimulant-Specific Overdose Risk Score Category [file jamanetwopen-e2113977-s001.pdf]

Supplemental Online Content

Emara AK, Santana D, Grits D, et al. Exploration of overdose risk score and postoperative complications and health care use after total knee arthroplasty. *JAMA Netw Open*. 2021;4(6):e2113977. doi:10.1001/jamanetworkopen.2021.13977

eTable 1. Multivariate Regression Demonstrating Odds of Length of Stay >2 Days by Overdose Risk Score Category

eTable 2. Multivariate Regression Demonstrating Odds of Nonhome Discharge Disposition by Overdose Risk Score Category

eTable 3. Multivariate Regression Demonstrating Odds of All-Cause 90-Day Readmission by Overdose Risk Score Category

eTable 4. Multivariate Regression Demonstrating Odds of All-Cause 90-Day Emergency Department Visits by Overdose Risk Score Category

eTable 5. Multivariate Regression Demonstrating Odds of Procedure-Related 90-Day Readmission by Overdose Risk Score Category

eTable 6. Multivariate Regression Demonstrating Odds of Non–Procedure-Related 90-Day Readmisison by Overdose Risk Score Category

eTable 7. Multivariate Regression Demonstrating Odds of Procedure-Related 90-Day Emergency Department Visits by Overdose Risk Score Category

eTable 8. Multivariate Regression Demonstrating Odds of Non–Procedure-Related 90-Day Emergency Department Visits by Overdose Risk Score Category

eTable 9. Multivariate Regression Demonstrating Odds of Pain-Related 90-Day Emergency Department Visits by Overdose Risk Score Category

eTable 10. Distribution of Baseline Determinants Among Overdose Risk Score <300 vs ≥300 Groups After Propensity Score Matching

eTable 11. Distribution of Included Individuals by Overdose Risk Score Category

eTable 12. Distribution of Preoperative Diagnoses Within Included Cohort

eTable 13. Distribution Individual Determinants and Outcomes by Opioid-Specific Overdose Risk Score Category

eTable 14. Distribution of Individual Determinants and Outcomes by Sedative-Specific Overdose Risk Score Category

eTable 15. Distribution of Individual Determinants and Outcomes by Stimulant-Specific Overdose Risk Score Category

This supplemental material has been provided by the authors to give readers additional information about their work.

© 2021 Emara AK et al. *JAMA Network Open.*

eTable 1. Multivariate Regression Demonstrating Odds of Length of Stay >2 Days per Overdose Risk Score Category

Accounting for age, sex, smoking status, race, BMI and baseline comorbidities.

**Predictor**	**Odds Ratio**	**95% CI**	**P value**
**Sex (reference: female)**			
**Male**	0.69	0.563 - 0.846	<0.001
**BMI (reference: normal BMI 18.5-24.9Kg/m^2)**			
**Underweight (<18.5Kg/m^2)**	3.394	0.265 - 43.411	0.347
**Overweight (25-29.9Kg/m^2)**	0.822	0.576 - 1.173	0.281
**Obese Class I (30-34.9Kg/m^2)**	0.839	0.587 - 1.2	0.337
**Obese Class II (35-39.9Kg/m^2)**	1.05	0.724 - 1.523	0.795
**Obese Class III (≥40Kg/m^2)**	1.579	1.078 - 2.314	0.019
**Age group (reference: 60-69 years)**			
**18-29**	0	0 - 8.76175519552715e+222	0.967
**30-39**	1.265	0.406 - 3.946	0.685
**40-49**	0.591	0.287 - 1.218	0.154
**50-59**	1.005	0.749 - 1.347	0.976
**70-79**	1.891	1.502 - 2.381	<0.001
**80-89**	4.77	3.462 - 6.573	<0.001
**90+**	11439453.108	0 - Inf	0.966
**Smoking Status (reference: never smoker)**			
**Quit >6 months**	1.274	1.04 - 1.561	0.019
**Quit <6 months**	1.064	0.59 - 1.919	0.837
**Current smoker**	1.312	0.912 - 1.887	0.144
**Race (reference: white)**			
**American Indian / Alaska Native**	8.615	2.193 - 33.839	0.002
**Asian**	0.963	0.349 - 2.654	0.941
**Black**	2.715	2.142 - 3.441	<0.001
**Multiracial / Multicultural**	0.729	0.301 - 1.764	0.483
**CCI category (reference: 0-2)**			
**3-4**	1.355	1.103 - 1.665	0.004
**5+**	3.268	2.179 - 4.901	<0.001
**NarxCare score category (reference: Zero)**			
**1-99**	0.996	0.771 - 1.288	0.978
**100-199**	0.881	0.658 - 1.179	0.394
**200-299**	0.937	0.662 - 1.326	0.714
**300-399**	2.027	1.456 - 2.821	<0.001
**400-499**	3.038	2.058 - 4.485	<0.001
**500+**	3.71	2.002 - 6.873	<0.001

OR: odds ratio; **CI**: confidence interval; **BMI**: body mass index; **CCI**: Charlson comorbidity index

© 2021 Emara AK et al. *JAMA Network Open.*

eTable 2. Multivariate Regression Demonstrating Odds of Nonhome Discharge Disposition per Overdose Risk Score Category

Accounting for age, sex, smoking status, race, BMI and baseline comorbidities.

**Predictor**	**Odds Ratio**	**95% CI**	**P value**
**Sex (reference: female)**			
**Male**	0.592	0.465 - 0.753	0.592
**BMI (reference: normal BMI 18.5-24.9Kg/m^2)**			
**Underweight (<18.5Kg/m^2)**	0	0 - Inf	0.986
**Overweight (25-29.9Kg/m^2)**	0.925	0.607 - 1.409	0.717
**Obese Class I (30-34.9Kg/m^2)**	0.979	0.639 - 1.498	0.921
**Obese Class II (35-39.9Kg/m^2)**	1.863	1.211 - 2.868	0.005
**Obese Class III (≥40Kg/m^2)**	2.009	1.274 - 3.169	0.003
**Age group (reference: 60-69 years)**			
**18-29**	0	0 - Inf	0.987
**30-39**	0.703	0.148 - 3.331	0.657
**40-49**	0.167	0.04 - 0.697	0.014
**50-59**	0.748	0.509 - 1.098	0.138
**70-79**	2.792	2.132 - 3.654	<0.001
**80-89**	9.375	6.586 - 13.346	<0.001
**90+**	154125077.578	0 - Inf	0.985
**Smoking Status (reference: never smoker)**			
**Quit >6 months**	0.861	0.677 - 1.093	0.219
**Quit <6 months**	0.55	0.243 - 1.246	0.152
**Current smoker**	1.598	1.063 - 2.401	0.024
**Race (reference: white)**			
**American Indian / Alaska Native**	0	0 - Inf	0.978
**Asian**	0.745	0.246 - 2.254	0.602
**Black**	2.421	1.84 - 3.185	<0.001
**Multiracial / Multicultural**	0.965	0.388 - 2.397	0.939
**CCI category (reference: 0-2)**			
**3-4**	1.254	0.989 - 1.588	0.061
**5+**	3.062	1.965 - 4.771	<0.001
**NarxCare score category (reference: Zero)**			
**1-99**	**0.975**	**0.724 - 1.312**	**0.865**
**100-199**	**1.061**	**0.768 - 1.466**	**0.721**
**200-299**	**0.765**	**0.498 - 1.174**	**0.221**
**300-399**	**2.006**	**1.371 - 2.937**	**<0.001**
**400-499**	**3.155**	**2.023 - 4.919**	**<0.001**
**500+**	**4.091**	**2.018 - 8.294**	**<0.001**

OR: odds ratio; **CI**: confidence interval; **BMI**: body mass index; **CCI**: Charlson comorbidity index

© 2021 Emara AK et al. *JAMA Network Open.*

eTable 3. Multivariate Regression Demonstrating Odds of All-Cause 90-Day Readmission per Overdose Risk Score Category

Accounting for age, sex, smoking status, race, BMI and baseline comorbidities.

Predictor	Odds Ratio	95% CI	P value
**Sex (reference: female)**			
**Male**	1.071	0.827 - 1.386	0.604
**BMI (reference: normal BMI 18.5-24.9Kg/m^2)**			
**Underweight (<18.5Kg/m^2)**	0	0 - Inf	0.981
**Overweight (25-29.9Kg/m^2)**	1.089	0.667 - 1.778	0.733
**Obese Class I (30-34.9Kg/m^2)**	0.868	0.524 - 1.437	0.583
**Obese Class II (35-39.9Kg/m^2)**	1.421	0.857 - 2.358	0.173
**Obese Class III (≥40Kg/m^2)**	1.42	0.829 - 2.431	0.201
**Age group (reference: 60-69 years)**			
**18-29**	0	0 - Inf	0.98
**30-39**	1.085	0.239 - 4.93	0.916
**40-49**	0.625	0.244 - 1.598	0.326
**50-59**	0.772	0.52 - 1.145	0.198
**70-79**	1.294	0.96 - 1.743	0.091
**80-89**	2.547	1.68 - 3.86	<0.001
**90+**	0	0 - Inf	0.986
**Smoking Status (reference: never smoker)**			
**Quit >6 months**	1.067	0.814 - 1.398	0.639
**Quit <6 months**	1.505	0.773 - 2.931	0.229
**Current smoker**	1.135	0.699 - 1.844	0.609
**Race (reference: white)**			
**American Indian / Alaska Native**	2.601	0.315 - 21.492	0.375
**Asian**	1.643	0.546 - 4.939	0.377
**Black**	1.855	1.345 - 2.558	<0.001
**Multiracial / Multicultural**	1.207	0.465 - 3.134	0.699
**CCI category (reference: 0-2)**			
**3-4**	1.468	1.12 - 1.923	0.005
**5+**	3.012	1.836 - 4.939	<0.001
**NarxCare score category (reference: Zero)**			
**1-99**	**0.959**	**0.688 - 1.337**	**0.806**
**100-199**	**0.776**	**0.524 - 1.147**	**0.204**
**200-299**	**0.623**	**0.373 - 1.04**	**0.07**
**300-399**	**1.563**	**1.01 - 2.421**	**0.045**
**400-499**	**2.036**	**1.224 - 3.387**	**0.006**
**500+**	**4.408**	**2.232 - 8.707**	**<0.001**

OR: odds ratio; **CI**: confidence interval; **BMI**: body mass index; **CCI**: Charlson comorbidity index

© 2021 Emara AK et al. *JAMA Network Open.*

eTable 4. Multivariate Regression Demonstrating Odds of All-Cause 90-Day Emergency Department Visits per Overdose Risk Score Category

Accounting for age, sex, smoking status, race, BMI and baseline comorbidities.

**Predictor**	**Odds Ratio**	**95% CI**	**P value**
**Sex (reference: female)**			
**Male**	0.909	0.734 - 1.125	0.381
**BMI (reference: normal BMI 18.5-24.9Kg/m^2)**			
**Underweight (<18.5Kg/m^2)**	0	0 - Inf	0.981
**Overweight (25-29.9Kg/m^2)**	1.113	0.744 - 1.664	0.602
**Obese Class I (30-34.9Kg/m^2)**	1.067	0.715 - 1.594	0.75
**Obese Class II (35-39.9Kg/m^2)**	0.993	0.651 - 1.515	0.975
**Obese Class III (≥40Kg/m^2)**	1.507	0.983 - 2.31	0.06
**Age group (reference: 60-69 years)**			
**18-29**	0	0 - Inf	0.979
**30-39**	10.154	4.282 - 24.075	<0.001
**40-49**	2.812	1.743 - 4.538	<0.001
**50-59**	1.409	1.059 - 1.874	0.019
**70-79**	1.364	1.062 - 1.753	0.015
**80-89**	1.304	0.853 - 1.995	0.22
**90+**	0	0 - Inf	0.985
**Smoking Status (reference: never smoker)**			
**Quit >6 months**	1.19	0.953 - 1.486	0.126
**Quit <6 months**	1.979	1.19 - 3.29	0.009
**Current smoker**	1.447	1.007 - 2.08	0.046
**Race (reference: white)**			
**American Indian / Alaska Native**	3.013	0.607 - 14.944	0.177
**Asian**	1.672	0.637 - 4.388	0.296
**Black**	1.576	1.202 - 2.067	0.001
**Multiracial / Multicultural**	0.602	0.216 - 1.68	0.333
**CCI category (reference: 0-2)**			
**3-4**	1.081	0.859 - 1.36	0.506
**5+**	1.612	0.981 - 2.649	0.06
**NarxCare score category (reference: Zero)**			
**1-99**	**1.478**	**1.124 - 1.942**	**0.005**
**100-199**	**1.424**	**1.056 - 1.92**	**0.02**
**200-299**	**0.949**	**0.643 - 1.399**	**0.79**
**300-399**	**1.62**	**1.105 - 2.375**	**0.013**
**400-499**	**2.035**	**1.288 - 3.214**	**0.002**
**500+**	**1.77**	**0.843 - 3.714**	**0.131**

OR: odds ratio; **CI**: confidence interval; **BMI**: body mass index; **CCI**: Charlson comorbidity index

© 2021 Emara AK et al. *JAMA Network Open.*

eTable 5. Multivariate Regression Demonstrating Odds of Procedure-Related 90-Day Readmission by Overdose Risk Score Category

Accounting for age, sex, smoking status, race, BMI and baseline comorbidities.

**Predictor**	**Odds Ratio**	**95% CI**	**P value**
**Sex (reference: female)**			
**Male**	1.351	0.884 - 2.065	0.165
**BMI (reference: normal BMI 18.5-24.9Kg/m^2)**			
**Underweight (<18.5Kg/m^2)**	0	0 - Inf	0.995
**Overweight (25-29.9Kg/m^2)**	1.863	0.71 - 4.888	0.206
**Obese Class I (30-34.9Kg/m^2)**	1.559	0.584 - 4.162	0.376
**Obese Class II (35-39.9Kg/m^2)**	1.553	0.56 - 4.301	0.397
**Obese Class III (≥40Kg/m^2)**	2.446	0.88 - 6.799	0.086
**Age group (reference: 60-69 years)**			
**18-29**	0	0 - Inf	0.995
**30-39**	7.103	1.837 - 27.465	0.004
**40-49**	0.813	0.186 - 3.552	0.783
**50-59**	1.336	0.745 - 2.395	0.331
**70-79**	1.12	0.654 - 1.92	0.68
**80-89**	2.743	1.403 - 5.361	0.003
**90+**	0	0 - Inf	0.996
**Smoking Status (reference: never smoker)**			
**Quit >6 months**	1.228	0.784 - 1.923	0.37
**Quit <6 months**	1.657	0.567 - 4.842	0.356
**Current smoker**	1.166	0.537 - 2.532	0.698
**Race (reference: white)**			
**American Indian / Alaska Native**	0	0 - Inf	0.992
**Asian**	2.627	0.574 - 12.029	0.214
**Black**	2.107	1.271 - 3.492	0.004
**Multiracial / Multicultural**	0	0 - Inf	0.977
**CCI category (reference: 0-2)**			
**3-4**	1.47	0.941 - 2.295	0.09
**5+**	2.219	0.94 - 5.238	0.069
**NarxCare score category (reference: Zero)**			
**1-99**	**1.126**	**0.656 - 1.932**	**0.668**
**100-199**	**0.61**	**0.294 - 1.264**	**0.184**
**200-299**	**0.708**	**0.307 - 1.635**	**0.419**
**300-399**	**1.458**	**0.692 - 3.071**	**0.321**
**400-499**	**1.838**	**0.78 - 4.335**	**0.164**
**500+**	**5.977**	**2.366 - 15.098**	**<0.001**

OR: odds ratio; CI: confidence interval; BMI: body mass index; CCI: Charlson comorbidity index

© 2021 Emara AK et al. *JAMA Network Open.*

eTable 6. Multivariate Regression Demonstrating Odds of Non–Procedure-Related 90-Day Readmisison by Overdose Risk Score Category

Accounting for age, sex, smoking status, race, BMI and baseline comorbidities.

**Predictor**	**Odds Ratio**	**95% CI**	**P value**
**Sex (reference: female)**			
**Male**	0.901	0.659 - 1.232	0.513
**BMI (reference: normal BMI 18.5-24.9Kg/m^2)**			
**Underweight (<18.5Kg/m^2)**	0	0 - Inf	0.992
**Overweight (25-29.9Kg/m^2)**	1.053	0.598 - 1.856	0.858
**Obese Class I (30-34.9Kg/m^2)**	0.724	0.4 - 1.311	0.286
**Obese Class II (35-39.9Kg/m^2)**	1.327	0.738 - 2.386	0.344
**Obese Class III (≥40Kg/m^2)**	1.276	0.682 - 2.388	0.446
**Age group (reference: 60-69 years)**			
**18-29**	0	0 - Inf	0.991
**30-39**	0	0 - Inf	0.978
**40-49**	0.521	0.159 - 1.712	0.283
**50-59**	0.558	0.334 - 0.931	0.026
**70-79**	1.264	0.894 - 1.788	0.185
**80-89**	2.083	1.262 - 3.439	0.004
**90+**	0	0 - Inf	0.994
**Smoking Status (reference: never smoker)**			
**Quit >6 months**	0.964	0.696 - 1.334	0.823
**Quit <6 months**	1.343	0.597 - 3.02	0.475
**Current smoker**	1.057	0.581 - 1.923	0.856
**Race (reference: white)**			
**American Indian / Alaska Native**	3.964	0.48 - 32.744	0.201
**Asian**	1.067	0.244 - 4.666	0.931
**Black**	1.832	1.25 - 2.685	0.002
**Multiracial / Multicultural**	1.78	0.686 - 4.62	0.236
**CCI category (reference: 0-2)**			
**3-4**	1.34	0.966 - 1.859	0.08
**5+**	2.999	1.701 - 5.288	<0.001
**NarxCare score category (reference: Zero)**			
**1-99**	**0.862**	**0.578 - 1.285**	**0.465**
**100-199**	**0.81**	**0.515 - 1.274**	**0.363**
**200-299**	**0.548**	**0.292 - 1.031**	**0.062**
**300-399**	**1.465**	**0.873 - 2.459**	**0.148**
**400-499**	**1.777**	**0.959 - 3.291**	**0.068**
**500+**	**2.663**	**1.071 - 6.623**	**0.035**

OR: odds ratio; **CI**: confidence interval; **BMI**: body mass index; **CCI**: Charlson comorbidity index

eTable 7. Multivariate Regression Demonstrating Odds of Procedure-Related 90-Day Emergency Department Visits by Overdose Risk Score Category

Accounting for age, sex, smoking status, race, BMI and baseline comorbidities.

**Predictor**	**Odds Ratio**	**95% CI**	**P value**
**Sex (reference: female)**			
**Male**	1.012	0.662 - 1.549	0.955
**BMI (reference: normal BMI 18.5-24.9Kg/m^2)**			
**Underweight (<18.5Kg/m^2)**	0	0 - Inf	0.996
**Overweight (25-29.9Kg/m^2)**	2.425	0.831 - 7.077	0.105
**Obese Class I (30-34.9Kg/m^2)**	2.624	0.906 - 7.603	0.076
**Obese Class II (35-39.9Kg/m^2)**	2.291	0.766 - 6.853	0.138
**Obese Class III (≥40Kg/m^2)**	3.476	1.153 - 10.483	0.027
**Age group (reference: 60-69 years)**			
**18-29**	0	0 - Inf	0.995
**30-39**	18.506	6.068 - 56.436	<0.001
**40-49**	2.04	0.75 - 5.548	0.163
**50-59**	1.428	0.804 - 2.536	0.224
**70-79**	1.555	0.936 - 2.582	0.088
**80-89**	1.414	0.599 - 3.34	0.429
**90+**	0	0 - Inf	0.996
**Smoking Status (reference: never smoker)**			
**Quit >6 months**	1.191	0.768 - 1.848	0.435
**Quit <6 months**	0.308	0.041 - 2.332	0.254
**Current smoker**	1.664	0.851 - 3.253	0.136
**Race (reference: white)**			
**American Indian / Alaska Native**	0	0 - Inf	0.992
**Asian**	1.333	0.175 - 10.142	0.781
**Black**	1.854	1.122 - 3.062	0.016
**Multiracial / Multicultural**	1.447	0.342 - 6.117	0.615
**CCI category (reference: 0-2)**			
**3-4**	0.803	0.499 - 1.291	0.365
**5+**	1.73	0.713 - 4.194	0.225
**NarxCare score category (reference: Zero)**			
**1-99**	**1.378**	**0.816 - 2.328**	**0.23**
**100-199**	**1.01**	**0.544 - 1.874**	**0.975**
**200-299**	**0.592**	**0.243 - 1.442**	**0.248**
**300-399**	**1.098**	**0.496 - 2.43**	**0.818**
**400-499**	**1.915**	**0.811 - 4.525**	**0.138**
**500+**	**3.545**	**1.196 - 10.51**	**0.022**

OR: odds ratio; **CI**: confidence interval; **BMI**: body mass index; **CCI**: Charlson comorbidity index

© 2021 Emara AK et al. *JAMA Network Open.*

Appendix 8. Multivariate Regression Demonstrating Odds of Non–Procedure-Related 90-Day Emergency Department Visits by Overdose Risk Score Category

Accounting for age, sex, smoking status, race, BMI and baseline comorbidities.

Predictor	Odds Ratio	95% CI	P value
**Sex (reference: female)**			
**Male**	0.717	0.541 - 0.949	0.02
**BMI (reference: normal BMI 18.5-24.9Kg/m^2)**			
**Underweight (<18.5Kg/m^2)**	0	0 - Inf	0.981
**Overweight (25-29.9Kg/m^2)**	1.094	0.668 - 1.791	0.721
**Obese Class I (30-34.9Kg/m^2)**	0.776	0.465 - 1.295	0.331
**Obese Class II (35-39.9Kg/m^2)**	1.238	0.74 - 2.072	0.417
**Obese Class III (≥40Kg/m^2)**	1.35	0.787 - 2.318	0.276
**Age group (reference: 60-69 years)**			
**18-29**	0	0 - Inf	0.98
**30-39**	0.549	0.071 - 4.229	0.565
**40-49**	1.161	0.537 - 2.511	0.704
**50-59**	0.691	0.449 - 1.063	0.093
**70-79**	1.373	1.009 - 1.869	0.044
**80-89**	2.395	1.542 - 3.72	<0.001
**90+**	0	0 - Inf	0.985
**Smoking Status (reference: never smoker)**			
**Quit >6 months**	1.05	0.79 - 1.394	0.739
**Quit <6 months**	1.725	0.889 - 3.346	0.107
**Current smoker**	1.187	0.718 - 1.962	0.505
**Race (reference: white)**			
**American Indian / Alaska Native**	2.606	0.318 - 21.323	0.372
**Asian**	0.774	0.178 - 3.36	0.733
**Black**	1.559	1.106 - 2.197	0.011
**Multiracial / Multicultural**	1.549	0.646 - 3.715	0.326
**CCI category (reference: 0-2)**			
**3-4**	1.394	1.05 - 1.851	0.022
**5+**	2.737	1.619 - 4.626	<0.001
**NarxCare score category (reference: Zero)**			
**1-99**	**1.043**	**0.734 - 1.482**	**0.815**
**100-199**	**0.943**	**0.633 - 1.405**	**0.773**
**200-299**	**0.861**	**0.526 - 1.409**	**0.551**
**300-399**	**1.727**	**1.095 - 2.723**	**0.019**
**400-499**	**2.072**	**1.203 - 3.567**	**0.009**
**500+**	**2.793**	**1.246 - 6.262**	**0.013**

OR: odds ratio; **CI**: confidence interval; **BMI**: body mass index; **CCI**: Charlson comorbidity index

eTable 9. Multivariate Regression Demonstrating Odds of Pain-Related 90-Day Emergency Department Visits by Overdose Risk Score Category

Accounting for age, sex, smoking status, race, BMI and baseline comorbidities.

**Predictor**	**Odds Ratio**	**95% CI**	**P value**
**Sex (reference: female)**			
**Male**	1.088	0.775 - 1.529	0.625
**BMI (reference: normal BMI 18.5-24.9Kg/m^2)**			
**Underweight (<18.5Kg/m^2)**	0	0 - Inf	0.989
**Overweight (25-29.9Kg/m^2)**	1.335	0.631 - 2.824	0.449
**Obese Class I (30-34.9Kg/m^2)**	1.264	0.597 - 2.677	0.541
**Obese Class II (35-39.9Kg/m^2)**	1.979	0.938 - 4.175	0.073
**Obese Class III (≥40Kg/m^2)**	2.475	1.153 - 5.315	0.02
**Age group (reference: 60-69 years)**			
**18-29**	0	0 - Inf	0.988
**30-39**	3.985	1.192 - 13.318	0.025
**40-49**	1.907	0.886 - 4.105	0.099
**50-59**	1.236	0.793 - 1.925	0.349
**70-79**	0.99	0.65 - 1.506	0.961
**80-89**	1.612	0.855 - 3.037	0.14
**90+**	0	0 - Inf	0.991
**Smoking Status (reference: never smoker)**			
**Quit >6 months**	1.352	0.943 - 1.939	0.101
**Quit <6 months**	1.499	0.625 - 3.592	0.364
**Current smoker**	1.894	1.107 - 3.24	0.02
**Race (reference: white)**			
**American Indian / Alaska Native**	4.539	0.531 - 38.804	0.167
**Asian**	2.898	0.831 - 10.106	0.095
**Black**	1.861	1.244 - 2.785	0.003
**Multiracial / Multicultural**	0.887	0.209 - 3.758	0.87
**CCI category (reference: 0-2)**			
**3-4**	1.007	0.692 - 1.466	0.971
**5+**	2.815	1.504 - 5.269	0.001
**NarxCare score category (reference: Zero)**			
**1-99**	**1.2**	**0.765 - 1.882**	**0.428**
**100-199**	**1.34**	**0.831 - 2.16**	**0.23**
**200-299**	**0.591**	**0.283 - 1.23**	**0.16**
**300-399**	**1.487**	**0.812 - 2.725**	**0.199**
**400-499**	**2.23**	**1.152 - 4.316**	**0.017**
**500+**	**4.89**	**2.123 - 11.262**	**<0.001**

© 2021 Emara AK et al. *JAMA Network Open.*

OR: odds ratio; **CI**: confidence interval; **BMI**: body mass index; **CCI**: Charlson comorbidity index

eTable 10. Distribution of Baseline Determinants Among Overdose Risk Score <300 vs ≥300 Groups After Propensity Score Matching

Baseline determinant	NCS<300 N(%)	NCS≥300 N(%)	p-value
**Cohort size**	2106	566	
**Sex (Female)**	1313 (62.3)	359 (63.4)	0.672
**BMI classification (%)**			0.873
**Overweight**	520 (24.7)	134 (23.7)	
**Normal weight**	208 (9.9)	58 (10.2)	
**Obese Class I**	593 (28.2)	156 (27.6)	
**Obese Class II**	461 (21.9)	121 (21.4)	
**Obese Class III**	324 (15.4)	97 (17.1)	
**Age (years)**			0.757
**30-39**	9 (0.4)	4 (0.7)	
**40-49**	60 (2.8)	18 (3.2)	
**50-59**	452 (21.5)	128 (22.6)	
**60-69**	920 (43.7)	254 (44.9)	
**70-79**	552 (26.2)	133 (23.5)	
**80-89**	113 (5.4)	29 (5.1)	
**Race**			0.976
**American Indian / Alaska Native**	2 (0.1)	1 (0.2)	
**Asian**	4 (0.2)	1 (0.2)	
**Black**	294 (14.0)	78 (13.8)	
**Multiracial / Multicultural**	39 (1.9)	13 (2.3)	
**White**	1726 (82.0)	461 (81.4)	
**CCI category**			0.778
**0-2**	1409 (66.9)	371 (65.5)	
**3-4**	634 (30.1)	179 (31.6)	
**5+**	63 (3.0)	16 (2.8)	

BMI: body mass index; **CCI**: charlson comorbidity index

© 2021 Emara AK et al. *JAMA Network Open.*

eTable 11. Distribution of Included Individuals by Overdose Risk Score Category

NarxCare Score category	Count (%)
**0**	1,440 (33.3)
**1-99**	1,066 (24.6)
**100-199**	769 (17.8)
**200-299**	476 (11.0)
**300-399**	327 (7.6)
**400-499**	185 (4.3)
**≥500**	63 (1.5)

© 2021 Emara AK et al. *JAMA Network Open.*

eTable 12. Distribution of Preoperative Diagnoses Within Included Cohort

**Diagnosis**	**Count (%)**
**Avascular necrosis**	22 (0.6%)
**Inflammatory arthritis**	28 (0.6%)
**Osteoarthritis**	4,170 (96.4%)
**Other**	21 (0.5%)
**Post-traumatic arthritis**	79 (1.8%)
**Tumor**	6 (0.1)
**Total**	4,326

© 2021 Emara AK et al. *JAMA Network Open.*

eTable 13. Distribution Individual Determinants and Outcomes by Opioid-Specific Overdose Risk Score Category

Variable	Opioid-Specific NarxCare Score							P-value
	Zero	1-99	100-199	200-299	300-399	400-499	500+	
**Number of patients**	N=1470	N=1142	N=849	N=426	N=251	N=153	N=35	
**Mean overall NarxCare Score (SD)**	2.76 (22.48)	61.94 (40.73)	167.43 (64.73)	262.72 (62.91)	362.85 (63.20)	457.35 (62.01)	579.40 (86.36)	**<0.001**
**Patients in each NarxCare Score Category**								**<0.001**
**0**	1440 (98.0%)	0 (0.0%)	0 (0.0%)	0 (0.0%)	0 (0.0%)	0 (0.0%)	0 (0.0%)	
**1-99**	14 (1.0%)	1052 (92.1%)	0 (0.0%)	0 (0.0%)	0 (0.0%)	0 (0.0%)	0 (0.0%)	
**100-199**	9 (0.6%)	69 (6.0%)	691 (81.4%)	0 (0.0%)	0 (0.0%)	0 (0.0%)	0 (0.0%)	
**200-299**	5 (0.3%)	15 (1.3%)	106 (12.5%)	350 (82.2%)	0 (0.0%)	0 (0.0%)	0 (0.0%)	
**300-399**	2 (0.1%)	6 (0.5%)	46 (5.4%)	54 (12.7%)	219 (87.3%)	0 (0.0%)	0 (0.0%)	
**400-499**	0 (0.0%)	0 (0.0%)	5 (0.6%)	17 (4.0%)	26 (10.4%)	137 (89.5%)	0 (0.0%)	
**500+**	0 (0.0%)	0 (0.0%)	1 (0.1%)	5 (1.2%)	6 (2.4%)	16 (10.5%)	35 (100.0%)	
**Age (mean (SD))**	67.37 (9.09)	66.98 (9.26)	66.53 (8.83)	65.75 (9.43)	65.02 (9.28)	63.78 (8.89)	58.77 (8.05)	**<0.001**
**Sex (Male (%))**	647 (44.0%)	434 (38.0%)	292 (34.4%)	162 (38.0%)	92 (36.7%)	69 (45.1%)	7 (20.0%)	**<0.001**
**BMI (mean (SD))**	32.43 (6.55)	32.51 (7.23)	33.23 (6.47%)	33.07 (7.99%)	34.29 (6.50%)	32.50 (7.40%)	33.90 (5.47)	**0.001**
**Smoking status (%)**								**<0.001**
**Non smoker**	888 (60.4%)	601 (52.6%)	472 (55.6%)	240 (56.3%)	123 (49.0%)	55 (35.9%)	14 (40.0%)	
**Quit >6 months**	473 (32.2%)	447 (39.1%)	295 (34.7%)	133 (31.2%)	89 (35.5%)	63 (41.2%)	9 (25.7%)	
**Quit <6 months**	38 (2.6%)	25 (2.2%)	27 (3.2%)	13 (3.1%)	6 (2.4%)	5 (3.3%)	2 (5.7%)	
**Current smoker**	71 (4.8%)	69 (6.0%)	55 (6.5%)	40 (9.4%)	33 (13.1%)	30 (19.6%)	10 (28.6%)	
**CCI (mean (SD))**	1.08 (1.59)	1.25 (1.72)	1.35 (1.80)	1.24 (1.69)	1.56 (1.71)	1.37 (1.70)	2.23 (1.88)	**<0.001**
**Length of stay (mean (SD))**	1.52 (1.12)	1.55 (1.09)	1.51 (0.92)	1.51 (0.98)	1.94 (2.26)	2.22 (2.02)	2.43 (2.38)	**<0.001**
**LOS >2 days**	171 (11.6%)	137 (12.0%)	102 (12.0%)	50 (11.7%)	49 (19.5%)	44 (28.8%)	11 (31.4%)	**<0.001**
**Non-home discharge**	130 (8.8%)	95 (8.3%)	82 (9.7%)	31 (7.3%)	40 (15.9%)	32 (20.9%)	8 (22.9%)	**<0.001**
**90-day readmission**	92 (6.3%)	74 (6.5%)	42 (4.9%)	18 (4.2%)	30 (12.0%)	20 (13.1%)	7 (20.0%)	**<0.001**
**Non-procedure-related 90-day readmission**	66 (4.5%)	48 (4.2%)	32 (3.8%)	10 (2.3%)	21 (8.4%)	11 (7.2%)	3 (8.6%)	**0.005**
**Procedure-related 90-day readmission**	30 (2.0%)	28 (2.5%)	10 (1.2%)	8 (1.9%)	9 (3.6%)	8 (5.2%)	4 (11.4%)	**<0.001**

© 2021 Emara AK et al. *JAMA Network Open.*

**90-day ED visit**	116 (7.9)	130 (11.4)	103 (12.1)	31 (7.3)	32 (12.7)	26 (17.0)	7 (20.0)	**<0.001**
**Non-procedure-related 90-day ED visits**	78 (5.3%)	66 (5.8%)	48 (5.7%)	17 (4.0%)	27 (10.8%)	16 (10.5%)	4 (11.4%)	**0.001**
**Procedure-related 90-day ED visits**	29 (2.0%)	34 (3.0%)	18 (2.1%)	3 (0.7%)	9 (3.6%)	7 (4.6%)	3 (8.6%)	**0.005**
**90-day reoperation**	12 (0.8%)	7 (0.6%)	1 (0.1%)	6 (1.4%)	3 (1.2%)	3 (2.0%)	1 (2.9%)	**0.044**
**Pain-related ED visit**	42 (2.9%)	45 (3.9%)	32 (3.8%)	6 (1.4%)	17 (6.8%)	13 (8.5%)	5 (14.3%)	**<0.001**

SD: standard deviation; **BMI**: body mass index; **CCI**: Charlson comorbidity index; **LOS**: length of stay; **ED**: emergency department

© 2021 Emara AK et al. *JAMA Network Open.*

eTable 14. Distribution of Individual Determinants and Outcomes by Sedative-Specific Overdose Risk Score Category

**Variable**	**Sedative-specific NarxCare Scores**							**p-value**
	**Zero**	**1-99**	**100-199**	**200-299**	**300-399**	**400-499**	**500+**	
**Number of patients**	**N=1470**	**N=1724**	**N=615**	**N=203**	**N=172**	**N=101**	**N=41**	
**Narx (mean (SD))**	2.76 (22.48)	90.04 (55.03)	256.89 (87.51)	287.02 (74.86)	367.84 (55.11)	457.38 (43.62)	614.39 (103.13)	**<0.001**
**Patients in each NarxCare Score Category**								**<0.001**
**0**	1440 (98.0%)	0 (0.0%)	0 (0.0%)	0 (0.0%)	0 (0.0%)	0 (0.0%)	0 (0.0%)	
**1-99**	14 (1.0%)	1052 (61.0%)	0 (0.0%)	0 (0.0%)	0 (0.0%)	0 (0.0%)	0 (0.0%)	
**100-199**	9 (0.6%)	603 (35.0%)	157 (25.5%)	0 (0.0%)	0 (0.0%)	0 (0.0%)	0 (0.0%)	
**200-299**	5 (0.3%)	64 (3.7%)	263 (42.8%)	144 (70.9%)	0 (0.0%)	0 (0.0%)	0 (0.0%)	
**300-399**	2 (0.1%)	5 (0.3%)	146 (23.7%)	33 (16.3%)	141 (82.0%)	0 (0.0%)	0 (0.0%)	
**400-499**	0 (0.0%)	0 (0.0%)	49 (8.0%)	24 (11.8%)	24 (14.0%)	88 (87.1%)	0 (0.0%)	
**500+**	0 (0.0%)	0 (0.0%)	0 (0.0%)	2 (1.0%)	7 (4.1%)	13 (12.9%)	41 (100.0)	
**Age (mean (SD))**	67.37 (9.09)	66.90 (9.05)	65.38 (9.59)	65.45 (9.74)	65.44 (8.43)	64.07 (7.44)	62.41 (11.02)	**<0.001**
**Sex (Male (%))**	647 (44.0%)	667 (38.7%)	225 (36.6%)	65 (32.0%)	57 (33.1%)	31 (30.7%)	11 (26.8%)	**<0.001**
**BMI (mean (SD))**	32.43 (6.55)	32.79 (7.06)	33.58 (7.42)	32.23 (5.89)	33.84 (7.49)	33.40 (7.10)	30.94 (5.01)	**0.002**
**Smoking status (%)**								**<0.001**
**Non smoker**	888 (60.4%)	916 (53.1%)	335 (54.5%)	96 (47.3%)	91 (52.9%)	44 (43.6%)	23 (56.1%)	
**Quit >6 months**	473 (32.2%)	653 (37.9%)	197 (32.0%)	83 (40.9%)	58 (33.7%)	36 (35.6%)	9 (22.0%)	
**Quit <6 months**	38 (2.6%)	42 (2.4%)	18 (2.9%)	9 (4.4%)	5 (2.9%)	2 (2.0%)	2 (4.9%)	
**Current smoker**	71 (4.8%)	113 (6.6%)	65 (10.6%)	15 (7.4%)	18 (10.5%)	19 (18.8%)	7 (17.1%)	
**CCI (mean (SD))**	1.08 (1.59)	1.29 (1.74)	1.29 (1.65)	1.30 (1.72)	1.69 (2.20)	1.32 (1.48)	1.61 (1.64)	**<0.001**
**Length of stay (mean (SD))**	1.52 (1.12)	1.52 (1.01)	1.62 (1.31)	1.67 (1.34)	1.81 (1.20)	2.28 (3.05)	2.41 (2.36)	**<0.001**
**LOS >2 days**	171 (11.6%)	198 (11.5%)	83 (13.5%)	35 (17.2%)	42 (24.4%)	23 (22.8%)	12 (29.3%)	**<0.001**
**Non-home discharge**	130 (8.8%)	158 (9.2%)	54 (8.8%)	24 (11.8%)	26 (15.1%)	18 (17.8%)	8 (19.5%)	**0.002**
**90-day readmission**	92 (6.3%)	97 (5.6%)	43 (7.0%)	14 (6.9%)	14 (8.1%)	13 (12.9%)	10 (24.4%)	**<0.001**

© 2021 Emara AK et al. *JAMA Network Open.*

**Non-procedure-related 90-day readmission**	66 (4.5%)	63 (3.7%)	29 (4.7%)	10 (4.9%)	9 (5.2%)	9 (8.9%)	5 (12.2%)	**0.034**
**Procedure-related 90-day readmission**	30 (2.0%)	36 (2.1%)	13 (2.1%)	4 (2.0%)	5 (2.9%)	4 (4.0%)	5 (12.2%)	**0.002**
**90-day ED visit**	116 (7.9%)	193 (11.2%)	58 (9.4%)	27 (13.3%)	32 (18.6%)	16 (15.8%)	3 (7.3%)	**<0.001**
**Non-procedure-related 90-day ED visits**	78 (5.3%)	90 (5.2%)	37 (6.0%)	14 (6.9%)	19 (11.0%)	12 (11.9%)	6 (14.6%)	**0.001**
**Procedure-related 90-day ED visits**	29 (2.0%)	45 (2.6%)	12 (2.0%)	5 (2.5%)	5 (2.9%)	4 (4.0%)	3 (7.3%)	0.275
**90-day reoperation**	12 (0.8%)	8 (0.5%)	7 (1.1%)	2 (1.0%)	1 (0.6%)	1 (1.0%)	2 (4.9%)	**0.048**
**Pain-related ED visit**	42 (2.9%)	64 (3.7%)	20 (3.3%)	7 (3.4%)	12 (7.0%)	9 (8.9%)	6 (14.6%)	**<0.001**

SD: standard deviation; **BMI**: body mass index; **CCI**: Charlson comorbidity index; **LOS**: length of stay; **ED**: emergency department

© 2021 Emara AK et al. *JAMA Network Open.*

eTable 15. Distribution of Individual Determinants and Outcomes by Stimulant-Specific Overdose Risk Score Category

Number of patients	Stimulant-specific NarxCare Scores					p-value
	Zero	1-99	100-199	200-299	300-399	
	N=4177	N=62	N=42	N=36	N=9	
**Narx (mean (SD))**	113.81 (136.99)	176.32 (148.09)	222.14 (146.14)	312.97 (124.28)	361.67 (146.74)	**<0.001**
**Patients in each NarxCare Score Category**						**<0.001**
**0**	1440 (34.5%)	0 (0.0%)	0 (0.0%)	0 (0.0%)	0 (0.0%)	
**1-99**	1040 (24.9%)	26 (41.9%)	0 (0.0%)	0 (0.0%)	0 (0.0%)	
**100-199**	724 (17.3%)	15 (24.2%)	30 (71.4%)	0 (0.0%)	0 (0.0%)	
**200-299**	437 (10.5%)	12 (19.4%)	2 (4.8%)	25 (69.4%)	0 (0.0%)	
**300-399**	305 (7.3%)	4 (6.5%)	5 (11.9%)	5 (13.9%)	8 (88.9%)	
**400-499**	177 (4.2%)	2 (3.2%)	2 (4.8%)	4 (11.1%)	0 (0.0%)	
**500+**	54 (1.3%)	3 (4.8%)	3 (7.1%)	2 (5.6%)	1 (11.1%)	
**Age (mean (SD))**	66.85 (9.11)	58.81 (9.13)	61.33 (8.16)	59.67 (8.22)	62.89 (5.37)	**<0.001**
**Sex (Male (%))**	1661 (39.8%)	15 (24.2%)	13 (31.0%)	11 (30.6%)	3 (33.3%)	0.066
**BMI (mean (SD)) Kg/m^2**	32.73 (6.92)	36.02 (7.07)	35.31 (5.83)	32.49 (5.25)	28.09 (3.86)	**<0.001**
**Smoking status (%)**						0.554
**Non smoker**	2309 (55.3%)	37 (59.7%)	24 (57.1%)	17 (47.2%)	6 (66.7%)	
**Quit >6 months**	1457 (34.9%)	17 (27.4%)	16 (38.1%)	17 (47.2%)	2 (22.2%)	
**Quit <6 months**	112 (2.7%)	3 (4.8%)	0 (0.0%)	0 (0.0%)	1 (11.1%)	
**Current smoker**	299 (7.2%)	5 (8.1%)	2 (4.8%)	2 (5.6%)	0 (0.0%)	
**CCI (mean (SD))**	1.24 (1.70)	1.05 (1.52)	0.93 (1.67)	1.36 (1.50)	1.89 (2.20)	0.446
**Length of stay (mean (SD))**	1.58 (1.24)	1.44 (0.80)	1.40 (0.73)	1.61 (0.73)	1.33 (0.71)	0.708
**LOS >2 days**	547 (13.1%)	7 (11.3%)	4 (9.5%)	5 (13.9%)	1 (11.1%)	0.953
**Non-home discharge**	409 (9.8%)	3 (4.8%)	6 (14.3%)	0 (0.0%)	0 (0.0%)	0.108
**90-day readmission**	275 (6.6%)	2 (3.2%)	5 (11.9%)	0 (0.0%)	1 (11.1%)	0.204
**Non-procedure-related 90-day readmission**	188 (4.5%)	2 (3.2%)	1 (2.4%)	0 (0.0%)	0 (0.0%)	0.597

© 2021 Emara AK et al. *JAMA Network Open.*

**Procedure-related 90-day readmission**	92 (2.2%)	0 (0.0%)	4 (9.5%)	0 (0.0%)	1 (11.1%)	**0.004**
**90-day ED visit**	435 (10.4%)	1 (1.6%)	5 (11.9%)	3 (8.3%)	1 (11.1%)	0.248
**Non-procedure-related 90-day ED visits**	249 (6.0%)	2 (3.2%)	3 (7.1%)	1 (2.8%)	1 (11.1%)	0.734
**Procedure-related 90-day ED visits**	102 (2.4%)	0 (0.0%)	0 (0.0%)	1 (2.8%)	0 (0.0%)	0.584
**90-day reoperation**	29 (0.7%)	0 (0.0%)	4 (9.5%)	0 (0.0%)	0 (0.0%)	**<0.001**
**Pain-related ED visit**	155 (3.7%)	0 (0.0%)	3 (7.1%)	1 (2.8%)	1 (11.1%)	0.262

SD: standard deviation; **BMI**: body mass index; **CCI**: Charlson comorbidity index; **LOS**: length of stay; **ED**: emergency department

© 2021 Emara AK et al. *JAMA Network Open.*
